# Involvement of the long intergenic non-coding RNA LINC00461 in schizophrenia

**DOI:** 10.1186/s12888-022-03718-4

**Published:** 2022-01-26

**Authors:** Shuquan Rao, Lin Tian, Hongbao Cao, Ancha Baranova, Fuquan Zhang

**Affiliations:** 1 State Key Laboratory of Experimental Hematology, National Clinical Research Center for Blood Diseases, Institute of Hematology & Blood Diseases Hospital, Chinese Academy of Medical Sciences & Peking Union Medical College, Tianjin, 300020 China; 2 Department of Psychiatry, Wuxi Mental Health Center of Nanjing Medical University, Wuxi, China; 3 School of Systems Biology, George Mason University (GMU), Fairfax, VA USA; 4 Research Centre for Medical Genetics, Moscow, 115478 Russia; 5 Department of Psychiatry, The Affiliated Brain Hospital of Nanjing Medical University, 264 Guangzhou Road, Nanjing, 210029 China

**Keywords:** Schizophrenia, fMRI, Hippocampus, LINC00461

## Abstract

**Objective:**

LINC00461 is a highly conserved intergenic non-protein coding RNA that was implicated in schizophrenia at the genome-wide level. We aim to explore potential mechanisms underlying the involvement of LINC00461 in schizophrenia.

**Methods:**

We performed a meta-analysis to investigate the association of LINC00461 rs410216 with schizophrenia, and evaluate the effects of the rs410216 on hippocampal volume and function using the functional magnetic resonance imaging (fMRI) analysis. We utilized the GTEx dataset to profile the expression distribution of LINC00461 across different brain regions, and to investigate the potential impact of the risk SNPs on the expression of LINC00461 and other nearby genes. We compared blood expression levels of LINC00461 between schizophrenia patients and controls.

**Results:**

Here we show that single-nucleotide polymorphisms (SNPs) located in regulatory elements spanning the LINC00461 region are significantly associated with schizophrenia (index SNP rs410216, P_meta_ = 1.43E-05); subjects carrying the risk allele of rs410216 showed decreased hippocampal volume. However, no significant association of the rs410216 variant with hippocampal activation was observed. Moreover, the expression level of LINC00461 mRNA was significantly lower in first-onset schizophrenia patients, and the risk allele also predicts a lower transcriptional level of LINC00461 in the hippocampus.

**Conclusion:**

Together, these convergent lines of evidence implicate inadequate LINC00461 expression in the hippocampus in the development of schizophrenia, providing novel insight into the genetic architecture and biological etiology of schizophrenia.

**Supplementary Information:**

The online version contains supplementary material available at 10.1186/s12888-022-03718-4.

## Introduction

Schizophrenia is a severe mental disorder with high heritability and strong genetic heterogeneity. Despite considerable heritability [1], the mechanism of schizophrenia remains elusive due to the phenotypic uncertainties and etiological heterogeneity. Accumulating studies point to structural and functional abnormalities in the hippocampus using imaging and postmortem studies of schizophrenia patients [2, 3]. In addition, hippocampal-dependent cognitive functions, such as memory, learning, and sensorimotor gating, are impaired in schizophrenia patients and are considered to be relevant to the severity and progress of the disorder [4].

Although protein-coding genes are cardinal forces influencing disease etiology, accumulating evidence strongly suggests that long noncoding RNA (lncRNA) may facilitate deciphering the disease pathogenesis [5]. Nevertheless, only a limited number of lncRNAs have been convincingly implicated in schizophrenia, i.e., GOMAFUI [5, 6]. The lncRNA LINC00461 is a feasible target in the pathogenesis of schizophrenia due to the following several aspects. First, LINC00461 is predominantly expressed in the brain (Supplementary Fig. 1). Second, the sequence and expression pattern of LINC00461 were highly conserved across diverse species [7], suggesting its crucial roles in the regulation of brain functions. Lastly, LINC00461 is one of the most pleiotropic genome-wide risk genes for major psychiatric traits [8], including schizophrenia [9].

In the present study, we report a significant genetic association of LINC00461 single nucleotide polymorphism (SNP) with schizophrenia. We also describe its association with several biological intermediate phenotypes, such as hippocampus structure, and cognitive performance. Furthermore, we reveal that the risk allele carriers predict lower mRNA levels of LINC00461, but not other nearby genes, in the hippocampus. These convergent results implicate LINC00461 in the biology of the hippocampus function and the pathophysiology of schizophrenia.

## Materials and methods

### Subjects

In the discovery stage, we used the statistical results from the largest schizophrenia GWAS conducted by the Schizophrenia Working Group of the Psychiatric Genomics Consortium (PGC2 schizophrenia GWAS) [10], which consisted of 49 ancestry-matched, non-overlapping case-control samples (46 of European and three of east Asian ancestry), and 3 family-based samples of European ancestry (totaling 35,476 cases and 46,839 matched controls). In detail, we extracted the data covering the LINC00461 gene locus, including 50 kb upstream and downstream regions in the present study.

Replication analyses were performed in three independent samples with no overlap with the discovery sample: 1) Chinese sample I from the Bio-X institutes of Shanghai Jiaotong University (7699 schizophrenia cases and 18,327 controls) [11]; 2) Chinese sample II from the Sixth Hospital, Peking University (4384 schizophrenia cases and 5770 controls) [12]; and 3) Jewish-Israeli family sample (107 nuclear families containing 331 individuals of whom 155 are affected) [13]. All the subjects provided written informed consent before their inclusion in the respective studies. All protocols used in the original studies reporting these samples were approved by the relevant Ethics Committee and followed all applicable institutional, national, and international guidelines. Most of these replication samples were previously reported in earlier GWAS or large-scale collaborative studies where they were found to be effective in detecting genetic risk variants for schizophrenia [14, 15]. Detailed sample description, genotyping method, quality control, genotype imputation, and statistical analysis can be found in the original manuscript and supplementary materials.

Linkage disequilibrium (LD) between rs410216 and the other studied SNPs was calculated using genotype data of CEU from the 1000 Genomes Project (Phase I). The online software, SNAP was used to plot the LD pattern [16].

### Functional magnetic resonance imaging (fMRI) analysis

Functional magnetic resonance images were obtained from 285 healthy German participants of European ancestry, as part of a tri-centric study (Mannheim, Bonn, and Berlin) on the neurogenetic mechanisms of psychiatric disease (the MooDS cohort) [17–19]. This particular experiment was approved by the ethics committees of the Universities of Bonn, Heidelberg, and Berlin.

During fMRI scanning, participants completed three consecutive memory tasks, including encoding, recall, and recognition of face profession, with an overall duration of 13 min, based on a paradigm previously used for imaging genetics [20]. Blood oxygen level-dependent fMRI was performed using two scanners (Siemens Trio 3 T; Siemens Medical Solutions, Erlangen, Germany) at the Central Institute of Mental Health Mannheim, the University of Bonn, and the Charité Universitätsmedizin, Berlin. fMRI images were processed using Statistical Parametric Mapping (SPM8).

A detailed description of the clinical samples, imaging parameters, functional imaging processing, and statistical analysis can be found in the original manuscript and the supplementary materials.

### Expression pattern of LINC00461 across different tissues and expression quantitative trait loci analysis

The GTEx (Genotype-Tissue Expression project) database contains information at the level of both genetic variation and gene expression from 53 human tissues (containing 13 brain regions) including different brain regions from 544 donors mainly of European ancestry [21]. We utilized this dataset to profile the expression distribution of LINC00461 across different brain regions, and to investigate the potential impact of the risk SNPs on the expression of LINC00461 and other nearby genes within 1 Mb.

### Analysis of LINC00461 expression by real-time quantitative PCR

For LINC00461 mRNA analysis in peripheral blood cells, blood was collected from 32 first-onset schizophrenia patients (15 males and 17 females, aged 38.563 ± 1.920 years ranging from 18 to 55 years) before and after 12-week treatment, and 48 healthy individuals (17 males and 31 females, aged 31.563 ± 0.992 years ranging from 21 to 45 years). The schizophrenia patients were antipsychotic drug-free for at least one month before enrollment. After completing baseline assessment, the schizophrenia patients were treated with oral atypical antipsychotics, including olanzapine (*n* = 10), quetiapine (*n* = 6), aripiprazole (n = 6), risperidone (*n* = 5), amisulpride (*n* = 3), and ziprasidone (*n* = 2). The study was approved by the Ethics Committee of the Wuxi Health Mental Center of Nanjing Medical University. All participants provided written informed consent before being enrolled in this study. Total RNA was isolated and the cDNA was synthesized following the manufacturers. RT-qPCR was performed using the primers of LINC00461: Forward 5′-TGGCGTGGACTACTCTGATG-3′; Reverse 5′-ACGTCCACCCAAGTGCTTAC-3′ in 7900HT real-time PCR machine (Applied Biosystems; USA) with ACTB as reference. The RT-qPCRs were performed in triplicate for each of the three independent samples.

The statistical analysis was performed by the GraphPad Prism 7 software (San Diego, CA, USA). Comparison between two groups was performed by the two-sided student’s t-test. The data are presented as mean ± SD. *P* < 0.05 was considered statistically significant.

## Results

### Common variants of LINC00461 confer risk for schizophrenia

**Fig. 1:**
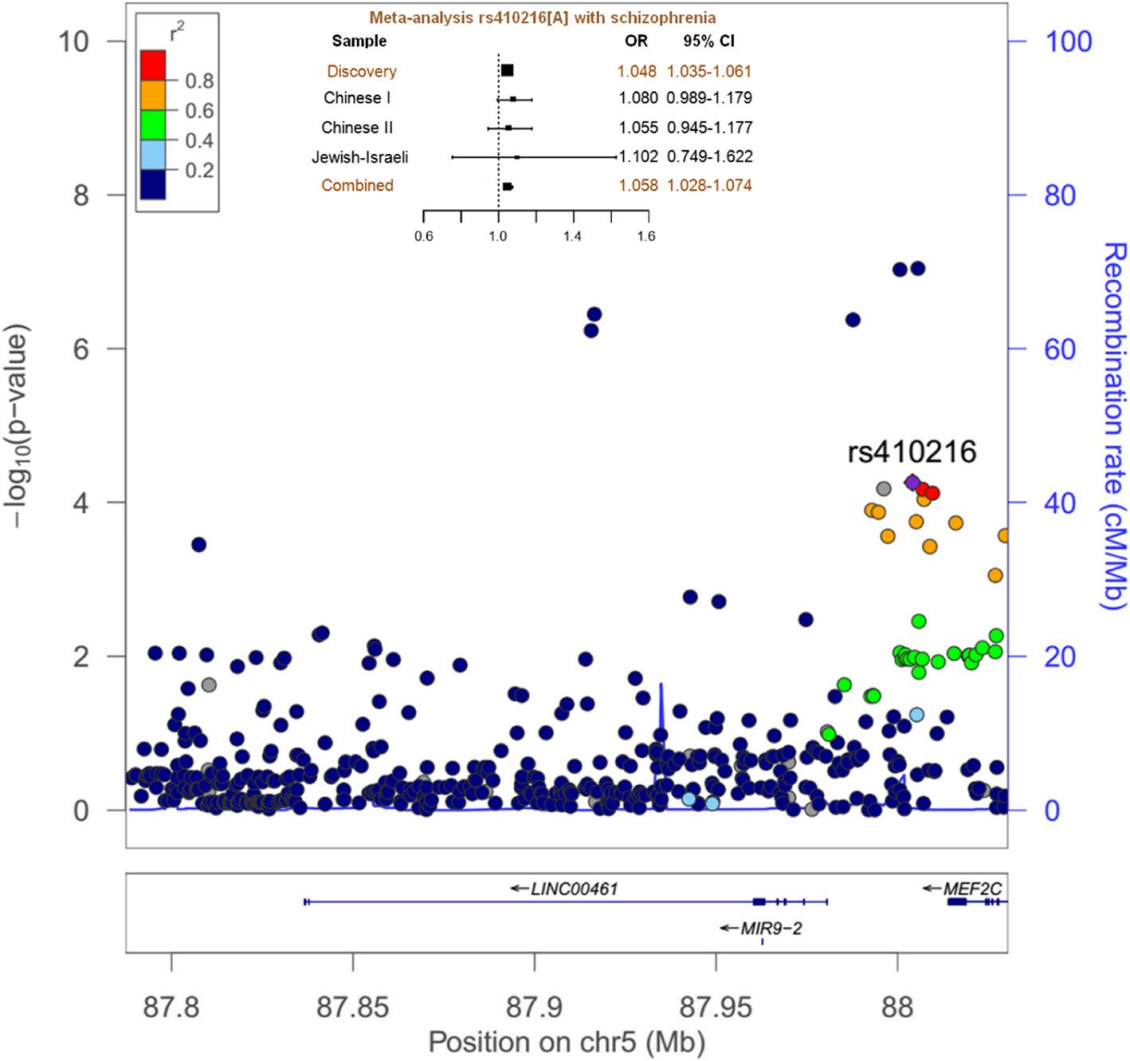
Genetic association of LINC00461variants with risk for schizophrenia. A physical map of genomic region (50 kb upstream and downstream regions covered) was given. SNPs showing region-wide significance of association with schizophrenia (*P*-value threshold, 0.05/494) was highlighted in red dots

Given that transcription regulatory elements can lie a considerable distance away from the gene locus, we retrieved the summary result of 494 SNPs covering both 50 kb upstream and downstream of the LINC00461 gene locus from the PGC2 schizophrenia GWAS [10]. Among the 494 SNPs, 10 SNPs showed region-wide association with schizophrenia, even after correction (P_Corrected_ < 0.05) (Fig. 1 and Supplementary Table 1). We excluded five SNPs (rs181900, rs254782, rs1644041, rs34960, and rs324899) due to: 1) minor allele frequency (MAF) less than 0.05 in European populations, and 2) none of them showed association with any of the intermediate phenotypes as revealed in the following analysis. Intriguingly, the remaining five SNPs showed moderate to strong LD with the index SNP, rs410216 (r^2^ = 0.56 ~ 1.00, in both European and Asian populations, the 1000 Genomes Pilot 1) (Supplementary Fig. 3) [22]. Therefore, we focused on rs410216 (with the lowest *P*-value among them) for further analysis.

To further confirm the observed association of rs410216 in PGC2 samples, we conducted replication analyses in three independent samples consisting of 12,083 patients and 24,097 controls, as well as 107 nuclear families (Supplementary Table 2). Significant association of rs410216 with schizophrenia was observed in these replication samples (risk allele A, P_replication_ = 0.024, OR = 1.07), with the effect in the same direction as that seen in the PGC2 samples. To increase the power of statistical association, we conducted a fixed-effect meta-analysis on the PGC2 and replication samples using the R package metafor. This meta-analysis showed that rs410216 was significantly associated with schizophrenia (risk allele A, P_meta_ = 1.43E-05, OR = 1.05), with no heterogeneity among individual samples (P_heterogeneity_ = 0.92, I^2^ = 0).

### Effects of the risk SNP on hippocampal volume and function

Subcortical brain regions can form circuits with cortical areas to coordinate learning, memory, cognition, and motivation whose dysfunctions have been frequently implicated in the neuropathology of schizophrenia [23]. We therefore hypothesized that the risk-associated SNPs could affect the biology of subcortical brain regions.

We first tested the effect of rs410216 on the structural variation of 7 subcortical regions (nucleus accumbens, caudate, putamen, pallidum, amygdala, hippocampus, and thalamus) using the ENIGMA 2 sample (*N* = 13,171) [24]. As shown in Supplementary Table 3, rs410216 was significantly associated with hippocampal volume (risk allele A; *P* = 0.014, β = − 12.10; β represents the difference in intracranial volume per copy increase of the risk allele), with the risk alleles predicting smaller hippocampal volume, while none of the other subcortical regions showed association with rs410216. Using another larger sample conducted by the ENIGMA and CHARGE consortium (*N* = 33,536, partially overlapping with ENIGMA 2 sample) [25], we further confirmed the significant association of rs410216 (*P* = 0.009) with hippocampal volume, with the effect in the same direction as that seen in ENIGMA 2 (Supplementary Table 4), providing strong evidence for its association with the hippocampus.

We next investigated the potential effect of different genotypes of rs410216 on hippocampal activation using fMRI. We conducted a region of interest analysis on 285 healthy control subjects of the MooDS cohort that completed 3 consecutive episodic memory tasks, including encoding, recall, and recognition of face-profession pairs [19]. However, no significant association of the rs410216 variant with hippocampal activation was observed for either hemisphere (family-wise error corrected *P* > 0.05, ROI) (Supplementary Table 5).

### The expression level of LINC00461 in schizophrenia patients and the association of rs410216 with LINC00461 expression

**Fig. 2:**
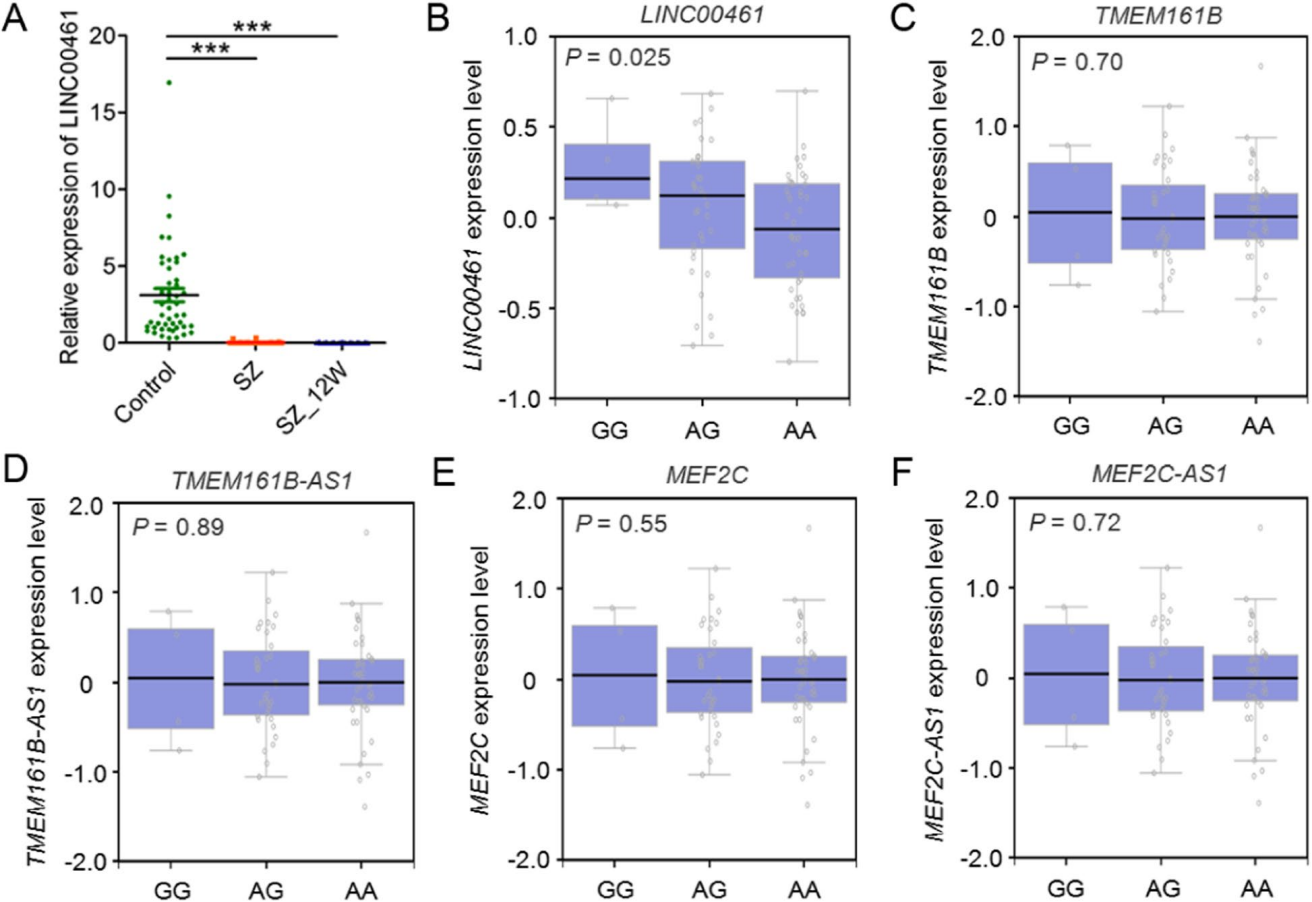
The expression level of LINC00461 in schizophrenia patients and regulated by rs410216 in the human hippocampus. **A** The LINC00461 mRNA level was decreased significantly in peripheral blood cells of first-onset schizophrenia (SZ) patients before and after 12-week treatment (SZ_12W) as compared with healthy controls (control *n* = 48, SZ *n* = 32, SZ_12W n = 32).***: P-value< 0.001 by Mann–Whitney u test. Data are presented as mean ± SEM. **B** rs410216 was significantly associated with the expression level of LINC00461 in the human hippocampus (*P* = 0.025). The individuals with AA genotype had lower LINC00461 expression level. **C**-**F** No evidence of association was observed between rs410216 with other nearby genes in 1 Mb genomic region, including TMEM161B, TMEM161B-AS1, MEF2C and MEF2C-AS1 (all P-value > 0.05). Data was retrieved from the GTEx. The Kruskal-Wallis test was used for the analysis

Real time-PCR revealed the expression level of LINC00461 mRNA was significantly lower in the peripheral blood cells of first-onset schizophrenia patients before and after 12-week treatment than in those of healthy individuals (Fig. 2A).

To test the effects of the risk SNPs onLINC00461 expression in vivo, we utilized GTEx [26]. As shown in Supplementary Fig. 4, LINC00461 is predominately expressed in brain tissues, such as the hippocampus, basal ganglia, and amygdala. Interestingly, we found that rs410216 showed a significant association with LINC00461 expression in the hippocampus (*P* = 0.025) but not in other brain regions (Fig. 2B and Supplementary Table 7). The risk allele carriers of rs410216 predicted reduced LINC00461 expression compared with the non-risk allele carriers. To further examine whether rs410216 was also associated with the expression of other nearby genes, we screened 4 genes (MEF2C, MEF2C-AS1, TMEM161B, and TMEM161B-AS1) in 1 Mb genomic region around rs410216. Interestingly, no significant association was observed between rs410216 and any of the four genes in the hippocampus (Fig. 2C-F).

Taken together, our data support the hypothesis that the gene dosage of LINC00461 might be down-regulated in schizophrenia and the mechanism by which the disease-associated SNPs contribute to risk for schizophrenia and related phenotypes involves the regulation of LINC00461 expression level and further suggest that dysfunction of the hippocampus would result in the biological effects related to the illness pathogenesis.

## Discussion

Here we reported a genetic association between the LINC00461 gene and schizophrenia susceptibility in European populations. We also examined whether rs410216 was also associated with educational attainment, a proxy phenotype of cognitive performance [27]. We found that rs410216 showed significant association with educational attainment (*P* = 6.03E-05), with the risk allele corresponding to decreased educational attainment (Supplementary Table 6) [28], supportive of the observation of an association between risk alleles and reduced hippocampal volume. Again, none of the five rare SNPs showed evidence of association with educational attainment (all *P* > 0.05) (Supplementary Table 6).

To move beyond the statistical association with a clinical diagnosis and to obtain convergent evidence of LINC00461 in the pathogenesis of schizophrenia, we have performed a series of convergent experiments testing the effects of risk SNPs on several intermediate biological phenotypes, involving hippocampal volume and cognitive function. Moreover, risk alleles have been found to predict lower expression of LINC00461 only in the hippocampus. Given the consistent observations between human and mice, the likelihood that the same risk-associated allele would predict by chance variation in each of investigated phenotypes across diverse samples and always in the direction of effect is remote.

Among the 7666 intergenic lncRNAs, LINC0461 was reported to be the most highly conserved [29], suggesting the functional importance of this lncRNA through evolution. LINC00461 affects the expression levels of its neighbor gene MEF2C, which plays a vital role in neurodevelopment relevant to mental disorders [30, 31]. A recent GWAS study indicated that LINC00461 was associated with educational attainment (together with MEF2C) and involved in decreased brain size, abnormal cerebral cortex morphology, and abnormal hippocampal mossy fiber morphology [28]. A recent cross-trait GWAS meta-analysis has identified LINC00461 as a pleiotropic gene for multiple mental disorders [32].

Our previous study indicated that knockdown of C130071C03Rik in mice (homologous to LINC00461) resulted in behavioral changes resembling symptoms in psychiatric traits, and knocking down of C130071C03Rik in mice embryos weakened neuron migration, supporting the potential role of LINC00461 during embryonic development [8]. Recently, multiple functional pathways of LINC00461 have been revealed [33–41].

There are several limitations in the present study, and we should be cautious in the interpretation of the results. First, the association *P*-values for rs410216 and the other two rare variants did not achieve the conventional genome-wide level of statistical significance. There must be numerous true susceptibility SNPs that do not yet surpass the genome-wide significance threshold, and an alternative way to identify them may be collecting convergent evidence from functional and animal studies. Secondly, due to the lack of schizophrenia patients in the fMRI analysis, it is unknown whether there is an association between rs410216 and hippocampal activation in schizophrenia. Third, although we observed a significant association between rs410216 and the LINC00461 mRNA expression in the hippocampus, it was not known whether the LINC00461 expression was changed in schizophrenia due to the absence of transcriptome data of the hippocampus. Further studies are necessary to validate this issue. Lastly, we cannot preclude the possibility that rs410216 may be just one association signal but not the true causative variant. A functional study on how the expression of LINC0461 is regulated by individual SNP across this region is warranted.

In conclusion, our results suggest LINC00461 to be a promising risk gene for schizophrenia, and implicate inadequate LINC00461 expression in the hippocampus in the development of the illness, providing novel insight into the genetic mechanism of schizophrenia.

## Supplementary Information

**Additional file 1: Supplementary Figure 1**. Expression of *LINC00461* across 27 different human organs and tissues. **Supplementary Figure 2**. Representation of genomic region covering the *LINC00461* gene locus (Human Genome Version 19, https://genome.ucsc.edu/). **Supplementary Figure 3**. LD plots between rs410216 and adjacent SNPs within 1 Mb genomic regions in CEU (upper panel) and CHB/JPT (lower panel) populations (http://www.broad.mit.edu/mpg/snap/). **Supplementary Figure 4**. Spatial expression profiling of *LINC00461*in 53 human tissues from the GTEx [17]. **Supplementary Table 1**. Association results of 10 SNPs spanning the *LINC00461* locus with schizophrenia from the PGC2 samples and their allele frequencies in three major populations. **Supplementary Table 2**. Association results of rs410216 with schizophrenia in each replication sample. **Supplementary Table 3**. Association of risk SNPs of *LINC00461* with 7 subcortical regions [12]. **Supplementary Table 4**. Replication of association between rs410216 and hippocampal volume (data from the ENIGMA-CHARGE sample, *N* = 33,536) [13]. **Supplementary Table 5**. Results of functional magnetic resonance for rs410216 during episodic memory processing. **Supplementary Table 6**. Effect of the risk SNPs on educational attainment [15]. **Supplementary Table 7**. Association analysis of rs410216 with the *LINC00461* expression in 10 brain regions.

## Data Availability

For access to the data in this paper, interested researchers may contact the corresponding author.
