# Supplementary material for: Involvement of the long intergenic non-coding RNA LINC00461 in schizophrenia
Source: BMC Psychiatry. 2022 Jan 26;22:59. doi: 10.1186/s12888-022-03718-4 (PMC8790831; doi:10.1186/s12888-022-03718-4)
Supplement: Supplementary file 1 — Additional file 1: Supplementary Figure 1. Expression of LINC00461 across 27 different human organs and tissues. Supplementary Figure 2. Representation of genomic region covering the LINC00461 gene locus (Human Genome Version 19, https://genome.ucsc.edu/). Supplementary Figure 3. LD plots between rs410216 and adjacent SNPs within 1 Mb genomic regions in CEU (upper panel) and CHB/JPT (lower panel) populations (http://www.broad.mit.edu/mpg/snap/). Supplementary Figure 4. Spatial expression profiling of LINC00461in 53 human tissues from the GTEx [17]. Supplementary Table 1. Association results of 10 SNPs spanning the LINC00461 locus with schizophrenia from the PGC2 samples and their allele frequencies in three major populations. Supplementary Table 2. Association results of rs410216 with schizophrenia in each replication sample. Supplementary Table 3. Association of risk SNPs of LINC00461 with 7 subcortical regions [12]. Supplementary Table 4. Replication of association between rs410216 and hippocampal volume (data from the ENIGMA-CHARGE sample, N = 33,536) [13]. Supplementary Table 5. Results of functional magnetic resonance for rs410216 during episodic memory processing. Supplementary Table 6. Effect of the risk SNPs on educational attainment [15]. Supplementary Table 7. Association analysis of rs410216 with the LINC00461 expression in 10 brain regions. [file 12888_2022_3718_MOESM1_ESM.docx]

**Supplementary File**

**Table of contents**

**1. Description of recruited subjects**

1.1. PGC2 schizophrenia samples

1.2. Chinese sample I from the Bio-X institutes of Shanghai Jiaotong University

1.3. Chinese sample II from the Sixth Hospital, Peking University

1.4. Jewish-Israeli family sample

**2. Detailed information of functional magnetic resonance imaging analysis**

2.1. Subjects and genotyping

2.2. Imaging parameters

2.3. Functional imaging processing and statistical analysis

**3. Association analysis of subcortical structure**

**4. Association analysis with cognitive performance anxiety status**

**5. Supplementary figures**

5.1. Supplementary Figure 1 Expression of *LINC00461* across 27 different human organs and tissues

5.2. Supplementary Figure 2 Representation of genomic region covering the *LINC00461* gene locus (Human Genome Version 19, https://genome.ucsc.edu/)

5.3. Supplementary Figure 3 LD plots between rs410216 and adjacent SNPs within 1 Mb genomic regions in CEU (upper panel) and CHB/JPT (lower panel) populations (<http://www.broad.mit.edu/mpg/snap/>)

5.4. Supplementary Figure 4 Spatial expression profiling of *LINC00461*in 53 human tissues from the GTEx

**6. Supplementary tables**

6.1. Supplementary Table 1 Association results of 10 SNPs spanning the *LINC00461* locus with schizophrenia from the PGC2 samples and their allele frequencies in three major populations

6.2. Supplementary Table 2 Association results of rs410216 with schizophrenia in each replication sample

6.3. Supplementary Table 3 Association of risk SNPs of *LINC00461* with 7 subcortical regions

6.4. Supplementary Table 4 Replication of association between rs410216 and hippocampal volume (data from the ENIGMA-CHARGE sample, *N* = 33,536)

6.5. Supplementary Table 5 Results of functional magnetic resonance for rs410216 during episodic memory processing

6.6. Supplementary Table 6 Effect of the risk SNPs on educational attainment

6.7. Supplementary Table 7 Association analysis of rs410216 with the *LINC00461* expression in 10 brain regions

**7. Supplementary references**

**1. Description of recruited subjects**

1.1. PGC2 schizophrenia samples

Recently, the Schizophrenia Working Group of the Psychiatric Genomics Consortium performed the largest schizophrenia (SCZ) GWAS, which consisted of 49 ancestry-matched, non-overlapping case-control samples (46 of European and three of east Asian ancestry), and 3 family-based samples of European ancestry (totaling 35,476 cases and 46,839 matched controls) [1]. The genotypes from each study were first processed by unified quality control procedures, followed by imputation of SNPs and insertion-deletions using the 1000 Genomes Project reference panel. In each sample, association testing was conducted using imputed marker dosages and principal components (PCs) to control for population stratification. The results were combined using an inverse-variance weighted fixed effects model. After quality
control (imputation INFO score ≥ 0.6, MAF ≥ 0.01, and successfully imputed in ≥ 20 samples), around 9.5 million variants were finally considered. More details about sample description and statistical procedures can be found in the original study [1].

1.2. Chinese sample I from the Bio-X institutes of Shanghai Jiaotong University

The sample consisted of 7,699 schizophrenia cases and 18,327 controls that were genotyped on different platforms: 4,175 cases and 10,470 controls genotyped with the Affymetrix Genome-Wide Human SNP Array 6.0 (SNP6.0); 2,472 cases and 5,928 controls genotyped with the Affymetrix Axiom Genome-Wide CHB1Array Plate (CHB1); and 1,052 cases and 1,929 controls genotyped with CHB1 or the Illumina 1M Array (1M) [2]. All cases of Chinese ancestry were inpatients or outpatients with a history of more than2 years of schizophrenia, who were recruited from mental-health centers in China, interviewed by two independent psychiatrists, and diagnosed according to the Diagnostic and Statistical Manual of Mental Disorders (DSM)-IV criteria. The controls were randomly selected from Chinese volunteers (from hospitals and a community survey) who were asked to reply to a written invitation to evaluate their medical histories. Lists of potential control subjects were screened for suitability as a volunteer by excluding subjects with major mental illnesses. Detailed sample description, genotyping method, quality control, genotype imputation, and statistical analysis can be found in the original manuscript [2].

1.3. Chinese sample II from the Sixth Hospital, Peking University

The sample consisted of 4,384 schizophrenia cases and 5,770 controls who were obtained from multiple collaborating hospitals in the Chinese Schizophrenia Collaboration Group [3]. Consensus diagnoses were performed by at least two experienced psychiatrists according to the criteria for schizophrenia from the DSM-IV. None of the subjects exhibited severe medical complications or other psychiatric disorders. All control individuals were clinically determined to be free of psychiatric disorders or family history of such disorders (including first-, second- and third-degree relatives). Genotyping of samples was conducted using Illumina (San Diego, CA, USA) Genome-Wide Arrays, including the Illumina HumanHap610-Quad Bead Chips, the Human660W-Quad Bead Chip, and the Illumina Human Omni Zhonghua Bead Chips. Other information was detailed in the original manuscript [3].

1.4. Jewish-Israeli family sample

The Jewish-Israeli family sample consisted of 107 nuclear families (331 individuals of whom155 are affected) [4]. Clinical evaluation included a semi-structured interview, the Schedule for Affective Disorders and Schizophrenia - Lifetime Version (SADS-L), a Family History Diagnostic Interview (FHRDC), and an assessment of medical records. Primary diagnoses were established by a best-estimate procedure according to Research Diagnostic Criteria (RDC). DNA was genotyped on the Human CNV-370 BeadArrays (Illumina, USA). Transmission disequilibrium test (TDT) for family samples with more than one offspring and various structures was used for association analysis of the main family sample. More detailed information can be found in the original manuscript.

**2. Detailed information of functional magnetic resonance imaging analysis**

2.1. Subjects and genotyping

Functional magnetic resonance images were obtained from 285 healthy German participants of European ancestry, as part of a tri-centric study on the neurogenetic mechanisms of psychiatric disease (the MooDS cohort) [5-7]. Exclusion criteria included a lifetime history of significant general medical, psychiatric or neurological illness, prior drug or alcohol abuse, head trauma, and the presence of a first-degree relative with mental illness. Heidelberg and Berlin. All subjects provided written informed consent to participate in the study.

2.2. Imaging parameters

Blood oxygen level-dependent fMRI was performed using 2 scanners (Siemens Trio 3T; Siemens Medical Solutions, Erlangen, Germany) at the Central Institute of Mental Health Mannheim, the University of Bonn, and the Universitätsmedizin Charité, Berlin. At these sites, the sequences and scanner protocols were identical and can be found in our previously published studies [5-7]. Quality assurance measures were conducted on every measurement day at all sites according to a multicenter quality assurance protocol revealing stable signals over time. To stringently account for any differences in signal-to-noise across sites, site was used as a covariate of no interest for all statistical analyses.

2.3. Functional imaging processing and statistical analysis

fMRI images were processed using Statistical Parametric Mapping (SPM8, <http://www.fil.ion.ucl.ac.uk/spm/>) and were similar for all tasks. The procedures followed our previously published studies [6, 8]. Briefly, images were realigned to a mean image (movement parameters were confined to <3-mm translation and<3° rotation between volumes), slice-time corrected, spatially normalized to standard stereotactic space (a brain template created by the Montreal Neurological Institute) with volume units (voxels) of 2 X 2 X 2 mm, and smoothed with an 8-mm full width-at-half-maximum gaussian filter; the ratio was normalized to the whole-brain global mean. A first-level fixed-effects model was computed for each participant. Regressors were created from the time course of the 2 experimental conditions (memory and control) and convolved with a canonical hemodynamic response function. Movement parameters were included in the first-level model as regressors of no interest. For each participant, statistical contrast images of memory vs control were obtained. To test for genetic association, these contrast images were analyzed using the multiple regression model including the three allelic groups (labeled as 0,1,2) as variables of interest and age, sex, and scanner site as the nuisance covariates. The significance threshold was set to *P*< 0.05, corrected for multiple comparisons across the whole brain. For all analyses, we used conservative analysis statistics by using the false discovery rate (FDR), a widely used method previously shown to exert strong control of type I error over multiple comparisons in imaging genetics [9]. The hippocampal ROI for seed voxel extraction and ROI analysis was defined a prior and created using anatomical labels provided by the Wake Forest University Pick Atlas [10].

**3. Association analysis of the subcortical structure**

Genetic variations exert lasting influences on brain structures associated with behavior and predisposition to the disease [11]. Recently, the ENIGMA2 consortium conducted a GWAS on the volumes of seven subcortical regions (nucleus accumbens, caudate, putamen, pallidum, amygdala, hippocampus, and thalamus) derived from magnetic resonance images (MRI) of 13,171 individuals [12]. More recently, a GWAS meta-analysis of mean bilateral hippocampal volume was performed. High-resolution MRI brain scans and genome-wide genotyping data were available for 33,536 individuals from 65 sites in two large consortia: the ENIGMA Consortium and the CHARGE Consortium [13]. In both GWASs, the additive dosage value of each SNP was regressed against the trait of interest separately using a multiple linear regression framework controlling for age, sex, four MDS components, ICV, and diagnosis (when applicable). The standardized protocols for image analysis, quality assessment, genetic imputation, and association can be found in the original manuscript [12, 13].

**4. Association analysis with cognitive performance**

We used educational attainment as a proxy phenotype for cognitive performance due to the positive correlation between educational attainment and cognitive ability (*r* ~ 0.5) [14], suggesting that educational attainments measured in large numbers of individuals can provide sufficient power to characterize the genetic influences of related phenotypes, including cognition and neuropsychiatric diseases. A recent GWAS meta-analysis was performed on 293,723 individuals from the UK Biobank, in which educational attainment is measured in all main analyses as the number of years of schooling completed (EduYears, mean= 14.3, s.d.= 3.6) [15]. All GWASs were performed at the cohort level in samples restricted to individuals of European descent whose educational attainment was assessed at or above age 30. The data of *LINC00461* SNPs were extracted. More details about sample description, genotyping, imputation, quality control, and statistic model were described previously [15].

**5. Supplementary figures**

5.1. Supplementary Figure 1 Expression of *LINC00461* across 27 different human organs and tissues


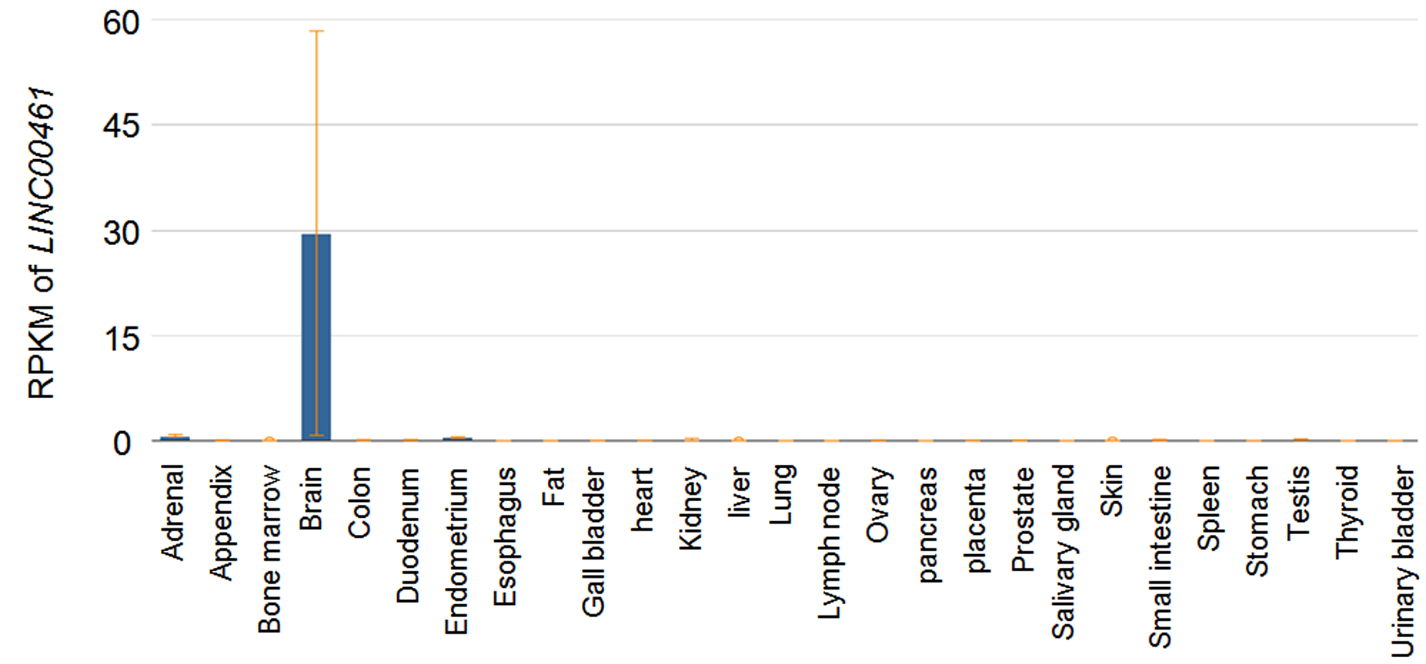


The transcriptome of 27 different human organs and tissues was analyzed using a next-generation sequencing-based on specimens from 95 individuals. These tissues were selected to represent tissue types with specialized body functions and to include all major organs and tissues. All tissues were microscopically examined to ensure the representation of normal tissue and to estimate the fraction of normal cell types in each sample. The transcriptome of each sample was quantified using RNA-Seq to determine the normalized mRNA abundance, calculated as FPKM-values. A detailed description of sample source, raw data acquisition, and data processing can be found in the original manuscript [16].

5.2. Supplementary Figure 2 Representation of genomic region covering the *LINC00461* gene locus (Human Genome Version 19, https://genome.ucsc.edu/)


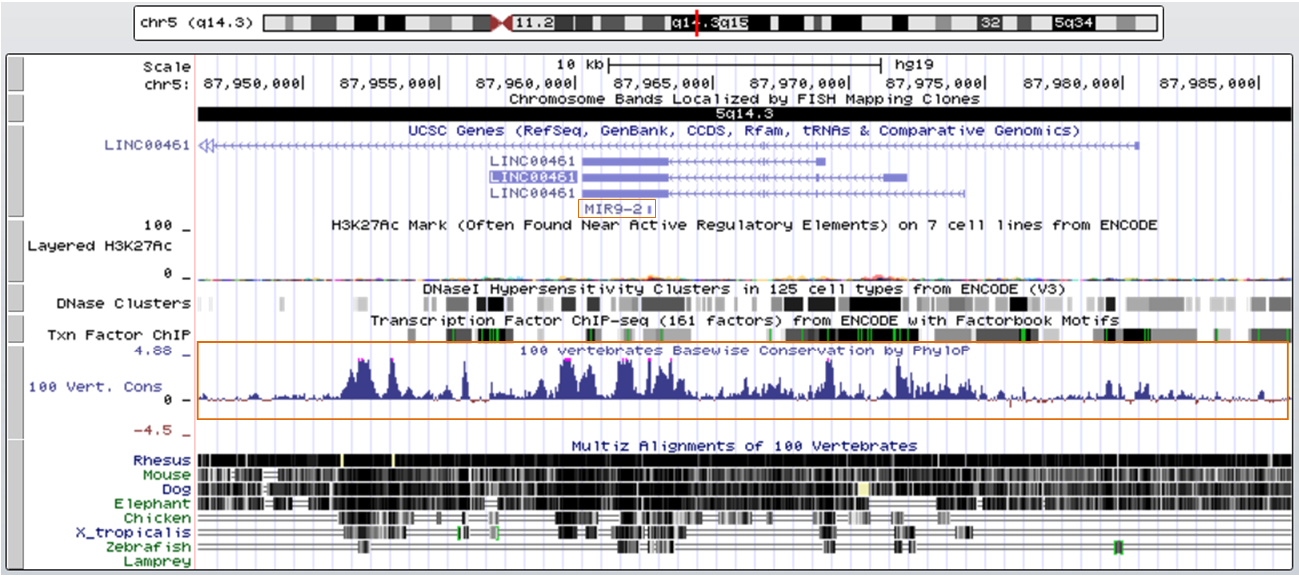


*MIR9-2*, highlighted in a red rectangular box, is located in the *LINC00461* gene locus.

The exon sequence of *LINC00461* is highly conserved across 100 vertebrates, as indicated by Phylop analysis (in a red rectangular box).

5.3. Supplementary Figure 3 LD plots between rs410216 and adjacent SNPs within 1 Mb genomic regions in CEU (upper panel) and CHB/JPT (lower panel) populations (<http://www.broad.mit.edu/mpg/snap/>)


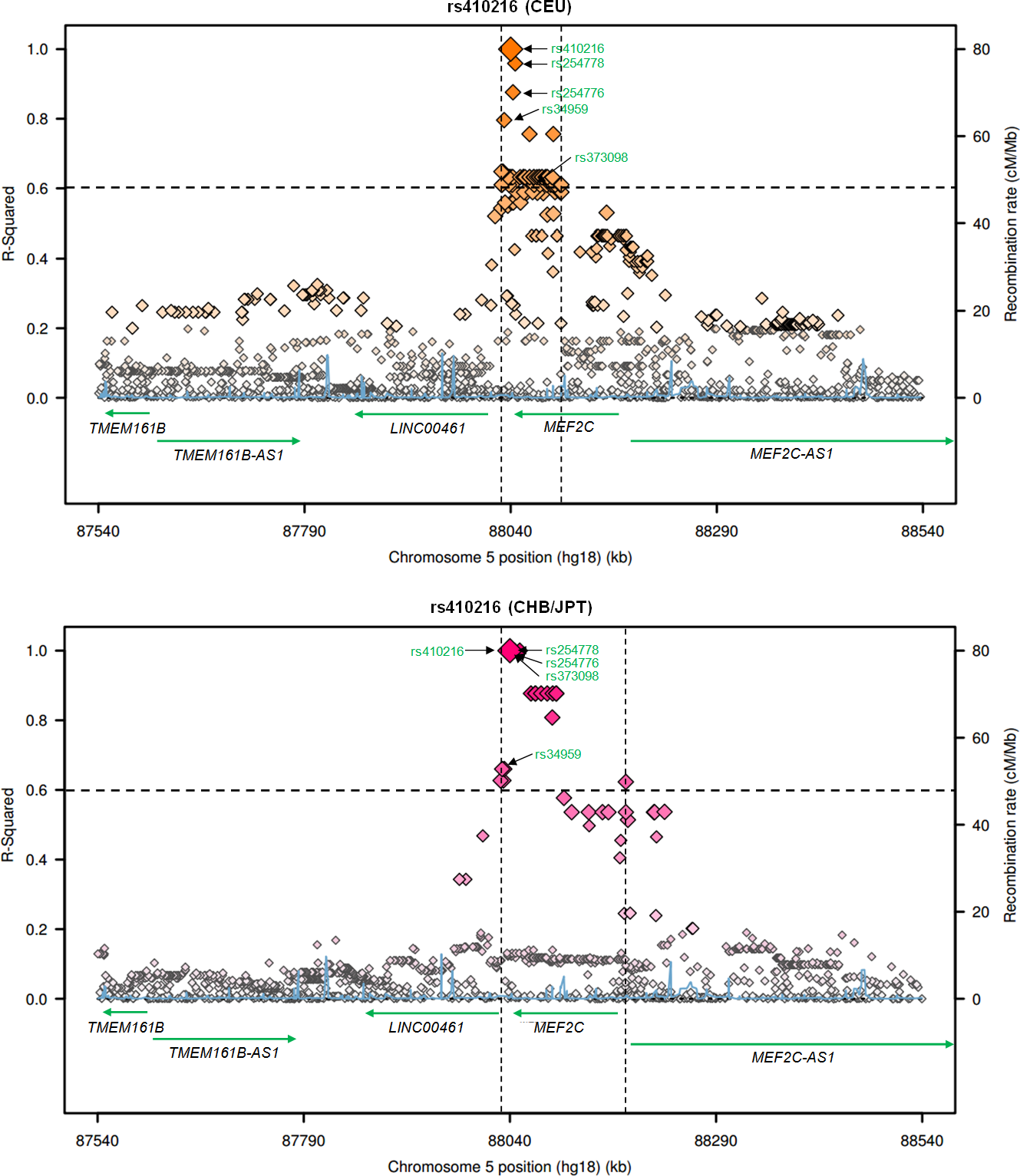


CEU, Utah Residents (CEPH) with Northern and Western European Ancestry; CHB, Han Chinese in Beijing, China; JPT, Japanese in Tokyo, Japan.

5.4. Supplementary Figure 4 Spatial expression profiling of *LINC00461*in 53 human tissues from the GTEx [17].


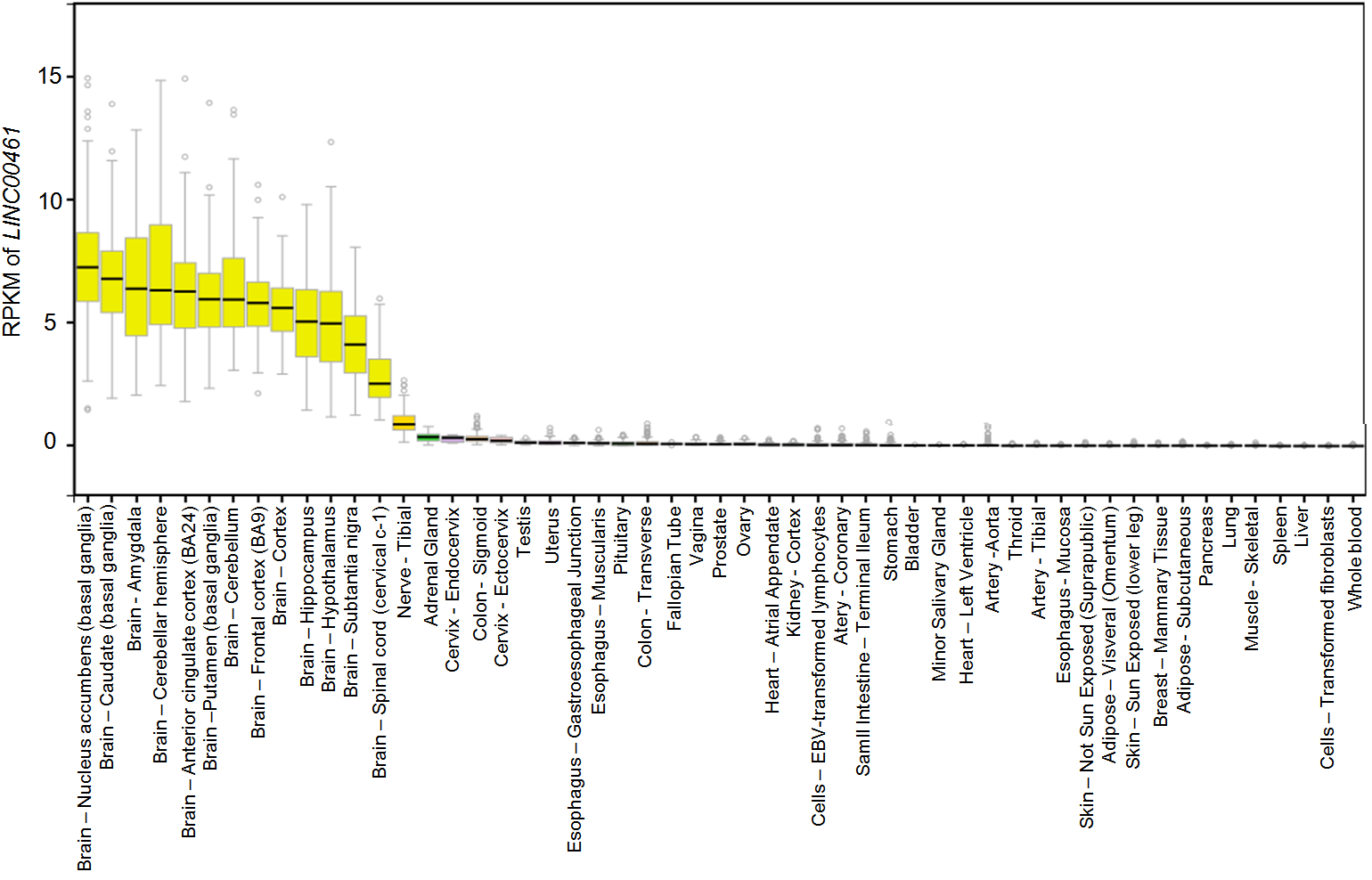


The GTEx database consisted of transcriptome data based on RNA-sequencing from 53 different tissues of 544 donors. The detailed information (*i.e.*, age, gender, and other clinic characteristics) of each individual can be found at <https://gtexportal.org>. Gene expression was presented as median reads per kilobase million (RPKM), and differentiated by gender, with females highlighted in red and males highlighted in blue.

**6. Supplementary tables**

6.1. Supplementary Table 1 Association results of 10 SNPs spanning the *LINC00461* locus with schizophrenia from the PGC2 samples and their allele frequencies in three major populations

| RSID | Pos. | Location | Allele1 | Allele2 | MAF | | | OR | S.E. | *P* | *P_Corrected_* |
| --- | --- | --- | --- | --- | --- | --- | --- | --- | --- | --- | --- |
|  |  |  |  |  | EAS | EUR | AFR |  |  |  |  |
| rs181900 | 88005568 | 5’neargene | A | C | 0 | 0.036 | 0.002 | 0.872 | 0.026 | 9.04E-08 | 4.46E-05 |
| rs254782 | 88000630 | 5’neargene | A | G | 0 | 0.036 | 0.002 | 0.872 | 0.026 | 9.37E-08 | 4.63E-05 |
| rs1644041 | 87916469 | intron | T | G | 0.005 | 0.032 | 0.003 | 0.879 | 0.025 | 3.57E-07 | 1.76E-04 |
| rs34960 | 87987689 | 5’neargene | A | G | 0 | 0.028 | 0.001 | 0.867 | 0.028 | 4.22E-07 | 2.09E-04 |
| rs324899 | 87915582 | Intron | A | G | 0.001 | 0.032 | 0.006 | 0.905 | 0.020 | 5.82E-07 | 2.88E-04 |
| rs410216 | 88004101 | 5’neargene | A | G | 0.064 | 0.314 | 0.483 | 1.048 | 0.012 | 5.52E-05 | 2.73E-02 |
| rs34959 | 87996170 | 5’neargene | C | G | 0.314 | 0.122 | 0.492 | 1.047 | 0.012 | 6.67E-05 | 3.30E-02 |
| rs254776 | 88006893 | 5’neargene | C | G | 0.064 | 0.311 | 0.488 | 1.047 | 0.012 | 6.80E-05 | 3.36E-02 |
| rs254778 | 88009593 | 5’neargene | A | G | 0.065 | 0.312 | 0.488 | 1.047 | 0.012 | 7.62E-05 | 3.76E-02 |
| rs373098 | 88007261 | 5’neargene | T | C | 0.064 | 0.260 | 0.461 | 1.049 | 0.012 | 9.17E-05 | 4.53E-02 |

The first five columns give the SNP identifier (RSID), genomic position (Pos.), location in the gene locus (location), Allele 1 and Allele 2 on Human Genome Version 19. The next three columns show the minor allele frequency (MAF) in three super populations based on the 1000 Genome Project. EAS, East Asian; EUR, European; AFR, African. The final four columns present the results of association from the PGC2 schizophrenia GWAS [1], including odds ratio (OR), standard error (S.E.), nominal *P*-value (*P*) and Bonferroni corrected *P*-value (*P*_Corrected_) based on allele 1.

6.2. Supplementary Table 2 Association results of rs410216 with schizophrenia in each replication sample

| Sample | Sample size | | Diagnostic criteria | Genotyping method | Risk allele | OR | S.E. | *P*-value |
| --- | --- | --- | --- | --- | --- | --- | --- | --- |
|  | **Case** | **Control** |  |  |  |  |  |  |
| Chinese I | 7,699 | 18,327 | DSM-IV | Affymetrix and Illumina | A | 1.080 | 0.045 | 0.087 |
| Chinese II | 4,384 | 5,770 | DSM-IV | Illumina | A | 1.055 | 0.056 | 0.340 |
| Jewish-Israeli families | 107 nuclear families | | SADS-L and FHRDC | Illumina | A | 1.102 | 0.197 | 0.622 |
| *Replication* | 12,083/24,097, 107 nuclear families | | -/- | -/- | A | 1.071 | 0.034 | 0.024 |

SADS-L, the Schedule for Affective Disorders and Schizophrenia-Lifetime Version; FHRDC, Family History Diagnostic Interview.

OR, odds ratio; S.E., standard error. In combined replication samples, a one-tailed *P*-value was shown.

6.3. Supplementary Table 3 Association of risk SNPs of *LINC00461* with 7 subcortical regions [12]

| SNP | Effect allele | Statistics | Thalamus | Putamen | Pallidum | Hippocampus | Caudate | Amygdala | Accumbens |
| --- | --- | --- | --- | --- | --- | --- | --- | --- | --- |
| rs181900 | A | *P*-value | 0.686 | 0.429 | 0.080 | 0.766 | 0.084 | 0.719 | 0.215 |
|  |  | β (S.E.) | 5.441(13.472) | -10.170(12.851) | -7.181(4.103) | 2.967(9.983) | -18.067(10.463) | 1.799(4.999) | -2.915(2.351) |
| rs254782 | A | *P*-value | 0.727 | 0.413 | 0.084 | 0.807 | 0.086 | 0.780 | 0.183 |
|  |  | β (S.E.) | 4.677(13.408) | -10.473(12.789) | -7.060(4.085) | 2.423(9.936) | -17.881(10.412) | 1.389(4.970) | -3.112(2.336) |
| rs1644041 | T | *P*-value | 0.825 | 0.252 | 0.062 | 0.943 | 0.075 | 0.667 | 0.344 |
|  |  | β (S.E.) | 3.050(13.787) | -15.042(13.138) | -7.906(4.236) | 0.735(10.264) | -19.019(10.697) | -2.218(5.161) | -2.306(2.437) |
| rs34960 | A | *P*-value | 0.502 | 0.705 | 0.218 | 0.635 | 0.151 | 0.480 | 0.215 |
|  |  | β (S.E.) | 9.937(14.814) | -5.351(14.128) | -5.561(4.515) | 5.219(10.994) | -16.545(11.522) | 3.886(5.505) | -3.246(2.615) |
| rs324899 | A | *P*-value | 0.609 | 0.442 | 0.092 | 0.644 | **0.024** | 0.622 | 0.170 |
|  |  | β (S.E.) | -6.020(11.765) | -8.640(11.234) | -6.098(3.616) | -4.087(8.847) | **-20.615(9.162)** | -2.197(4.454) | -2.877(2.095) |
| rs410216 | A | *P*-value | 0.093 | 0.600 | 0.635 | **0.014** | 0.954 | 0.115 | 0.364 |
|  |  | β (S.E.) | -11.107(6.622) | 3.321(6.332) | -0.959(2.022) | **-12.097(4.945)** | -0.295(5.158) | -3.937(2.496) | -1.065(1.173) |

Effect sizes (β) are given in units of mm^3^ per effect allele. Results are provided for the discovery samples (all European ancestry). For each SNP, the additive dosage value was regressed against the trait of interest separately using a multiple linear regression framework controlling for age, age^2^, sex, 4 MDS components, ICV (for non-ICV phenotypes), and diagnosis (when applicable). S.E., standard error.

**Note:** Although rs324899 showed significant association with caudate volume (*P* = 0.024, *β* = -20.62), the risk allele A of rs324899 predicted increased caudate volume, which was contradictory to the previous report of reduction in caudate volume in antipsychotic-naïve schizophrenia patients [18], implying that the observed association of rs324899 might have been generated by chance. We therefore didn’t focus on it in the following analysis.

6.4. Supplementary Table 4 Replication of association between rs410216 and hippocampal volume (data from the ENIGMA-CHARGE sample, *N* = 33,536) [13]

| SNP | Effect allele | *P*-value | Effect size | S.E. |
| --- | --- | --- | --- | --- |
| rs410216 | A | 0.009 | -2.616 | 0.001 |

The additive dosage value of rs410216 on mean bilateral hippocampal volume ((left + right)/2) was calculated after controlling for 4 MDS components, age, age^2^, sex, intracranial volume, and diagnosis (when applicable). S.E., standard error of effect; *P*-value, nominal *P*-value of the null hypothesis that the coefficient is equal to zero.

6.5. Supplementary Table 5 Results of functional magnetic resonance for rs410216 during episodic memory processing

| Tasks | Left hippocampus | | | | Right hippocampus | | | |
| --- | --- | --- | --- | --- | --- | --- | --- | --- |
|  | (x, y, z) | *P* | *Z* | *T* | (x, y, z) | *P* | *Z* | *T* |
| Encoding | (-21, -10, -20) | 0.82 | 1.54 | 1.53 | (21, -10, -20) | 0.90 | 1.19 | 1.16 |
| Recall | (-15, -10, -17) | 0.78 | 1.23 | 1.59 | (15, -10, -17) | 0.92 | 1.23 | 1.20 |
| Recognition | (-21, -28, -8) | 0.53 | 2.08 | 2.09 | (21, -28, -8) | 0.93 | 1.11 | 1.06 |

All results were corrected for multiple comparisons across the whole brain at *P* < 0.05 false discovery rate; x, y, and z, respective coordinates in the Montreal Neurological Institute template. *P*, *P*-value; *Z*, *Z*-value.

| SNP | Effect allele | Beta | S.E. | *P*-value |
| --- | --- | --- | --- | --- |
| rs181900 | A | -0.004 | 0.005 | 0.4302 |
| rs254782 | A | -0.003 | 0.005 | 0.574 |
| rs1644041 | T | -0.003 | 0.005 | 0.5881 |
| rs34960 | A | -0.007 | 0.006 | 0.216 |
| rs324899 | A | -0.001 | 0.005 | 0.7817 |
| rs410216 | A | -0.011 | 0.003 | 6.03E-05 |

6.6. Supplementary Table 6 Effect of the risk SNPs on educational attainment [15]

Beta, standardized regression coefficient; S.E., standard error of effect; *P*-value, nominal *P*-value of the null hypothesis that the coefficient is equal to zero.

6.7. Supplementary Table 7 Association analysis of rs410216 with the *LINC00461* expression in 10 brain regions

| **Gene** | **SNP** | **Effect allele** | ***P*-value** | **Effect size** | ***T*** | **S.E.** | **Tissue** |
| --- | --- | --- | --- | --- | --- | --- | --- |
| *LINC00461* | rs410216 | G | 0.56 | -0.047 | -0.58 | 0.08 | [Brain-Anterior cingulate cortex(BA24)](javascript:portalClient.eqtl.goTissuePage('Brain_Anterior_cingulate_cortex_BA24')) |
| *LINC00461* | rs410216 | G | 0.42 | -0.055 | -0.81 | 0.067 | [Brain-Caudate (basal ganglia)](javascript:portalClient.eqtl.goTissuePage('Brain_Caudate_basal_ganglia')) |
| *LINC00461* | rs410216 | G | 0.06 | -0.21 | -1.9 | 0.11 | [Brain-Cerebellar Hemisphere](javascript:portalClient.eqtl.goTissuePage('Brain_Cerebellar_Hemisphere')) |
| *LINC00461* | rs410216 | G | 0.38 | -0.092 | -0.89 | 0.1 | [Brain-Cerebellum](javascript:portalClient.eqtl.goTissuePage('Brain_Cerebellum')) |
| *LINC00461* | rs410216 | G | 0.5 | -0.057 | -0.68 | 0.084 | [Brain-Cortex](javascript:portalClient.eqtl.goTissuePage('Brain_Cortex')) |
| ***LINC00461*** | **rs410216** | **G** | **0.96** | **0.0038** | **0.055** | **0.069** | [**Brain-Frontal Cortex (BA9)**](javascript:portalClient.eqtl.goTissuePage('Brain_Frontal_Cortex_BA9')) |
| *LINC00461* | rs410216 | G | 0.025 | -0.19 | -2.3 | 0.084 | [Brain-Hippocampus](javascript:portalClient.eqtl.goTissuePage('Brain_Hippocampus')) |
| *LINC00461* | rs410216 | G | 0.4 | -0.071 | -0.84 | 0.085 | [Brain-Hypothalamus](javascript:portalClient.eqtl.goTissuePage('Brain_Hypothalamus')) |
| *LINC00461* | rs410216 | G | 0.099 | -0.1 | -1.7 | 0.062 | [Brain-Nucleus accumbens (basal ganglia)](javascript:portalClient.eqtl.goTissuePage('Brain_Nucleus_accumbens_basal_ganglia')) |
| *LINC00461* | rs410216 | G | 0.06 | -0.12 | -1.9 | 0.065 | [Brain-Putamen (basal ganglia)](javascript:portalClient.eqtl.goTissuePage('Brain_Putamen_basal_ganglia')) |

Data were extracted from the GTEx, ([www.gtexportal.org/](http://www.gtexportal.org/)) [17]. T, T-statistics; S.E., standard error.

**7. Supplementary references**

1. Schizophrenia Working Group of the Psychiatric Genomics C. Biological insights from 108 schizophrenia-associated genetic loci. Nature. 2014;511(7510):421-7.

2. Li Z, Chen J, Yu H, He L, Xu Y, Zhang D, Yi Q, Li C, Li X, Shen J et al. Genome-wide association analysis identifies 30 new susceptibility loci for schizophrenia. Nat Genet. 2017;49(11):1576-83.

3. Yu H, Yan H, Li J, Li Z, Zhang X, Ma Y, Mei L, Liu C, Cai L, Wang Q et al. Common variants on 2p16.1, 6p22.1 and 10q24.32 are associated with schizophrenia in Han Chinese population. Mol Psychiatry. 2017;22(7):954-60.

4. Alkelai A, Lupoli S, Greenbaum L, Kohn Y, Kanyas-Sarner K, Ben-Asher E, Lancet D, Macciardi F, Lerer B. DOCK4 and CEACAM21 as novel schizophrenia candidate genes in the Jewish population. Int J Neuropsychopharmacol. 2012;15(4):459-69.

5. Walter H, Schnell K, Erk S, Arnold C, Kirsch P, Esslinger C, Mier D, Schmitgen MM, Rietschel M, Witt SH et al. Effects of a genome-wide supported psychosis risk variant on neural activation during a theory-of-mind task. Mol Psychiatry. 2011;16(4):462-70.

6. Esslinger C, Walter H, Kirsch P, Erk S, Schnell K, Arnold C, Haddad L, Mier D, Opitz von Boberfeld C, Raab K et al. Neural mechanisms of a genome-wide supported psychosis variant. Science. 2009;324(5927):605.

7. Erk S, Meyer-Lindenberg A, Schnell K, Opitz von Boberfeld C, Esslinger C, Kirsch P, Grimm O, Arnold C, Haddad L, Witt SH et al. Brain function in carriers of a genome-wide supported bipolar disorder variant. Arch Gen Psychiatry. 2010;67(8):803-11.

8. Cao H, Plichta MM, Schafer A, Haddad L, Grimm O, Schneider M, Esslinger C, Kirsch P, Meyer-Lindenberg A, Tost H. Test-retest reliability of fMRI-based graph theoretical properties during working memory, emotion processing, and resting state. Neuroimage. 2014;84:888-900.

9. Meyer-Lindenberg A, Nicodemus KK, Egan MF, Callicott JH, Mattay V, Weinberger DR. False positives in imaging genetics. Neuroimage. 2008;40(2):655-61.

10. Maldjian JA, Laurienti PJ, Kraft RA, Burdette JH. An automated method for neuroanatomic and cytoarchitectonic atlas-based interrogation of fMRI data sets. Neuroimage. 2003;19(3):1233-9.

11. Blokland GA, de Zubicaray GI, McMahon KL, Wright MJ. Genetic and environmental influences on neuroimaging phenotypes: a meta-analytical perspective on twin imaging studies. Twin Res Hum Genet. 2012;15(3):351-71.

12. Hibar DP, Stein JL, Renteria ME, Arias-Vasquez A, Desrivieres S, Jahanshad N, Toro R, Wittfeld K, Abramovic L, Andersson M et al. Common genetic variants influence human subcortical brain structures. Nature. 2015;520(7546):224-9.

13. Hibar DP, Adams HHH, Jahanshad N, Chauhan G, Stein JL, Hofer E, Renteria ME, Bis JC, Arias-Vasquez A, Ikram MK et al. Novel genetic loci associated with hippocampal volume. Nat Commun. 2017;8:13624.

14. Rietveld CA, Medland SE, Derringer J, Yang J, Esko T, Martin NW, Westra HJ, Shakhbazov K, Abdellaoui A, Agrawal A et al. GWAS of 126,559 individuals identifies genetic variants associated with educational attainment. Science. 2013;340(6139):1467-71.

15. Okbay A, Beauchamp JP, Fontana MA, Lee JJ, Pers TH, Rietveld CA, Turley P, Chen GB, Emilsson V, Meddens SF et al. Genome-wide association study identifies 74 loci associated with educational attainment. Nature. 2016;533(7604):539-42.

16. Fagerberg L, Hallstrom BM, Oksvold P, Kampf C, Djureinovic D, Odeberg J, Habuka M, Tahmasebpoor S, Danielsson A, Edlund K et al. Analysis of the human tissue-specific expression by genome-wide integration of transcriptomics and antibody-based proteomics. Mol Cell Proteomics. 2014;13(2):397-406.

17. Consortium GT. Human genomics. The Genotype-Tissue Expression (GTEx) pilot analysis: multitissue gene regulation in humans. Science. 2015;348(6235):648-60.

18. Levitt JJ, McCarley RW, Dickey CC, Voglmaier MM, Niznikiewicz MA, Seidman LJ, Hirayasu Y, Ciszewski AA, Kikinis R, Jolesz FA et al. MRI study of caudate nucleus volume and its cognitive correlates in neuroleptic-naive patients with schizotypal personality disorder. Am J Psychiatry. 2002;159(7):1190-7.
